# Waterline Disinfectants Reduce Dental Bioaerosols: A Multitracer Validation

**DOI:** 10.1177/00220345221093522

**Published:** 2022-05-01

**Authors:** J.R. Allison, C. Dowson, N.S. Jakubovics, C. Nile, J. Durham, R. Holliday

**Affiliations:** 1 School of Dental Sciences, Faculty of Medical Sciences, Newcastle University, Newcastle upon Tyne, Tyne and Wear, UK; 2 Newcastle upon Tyne Hospitals NHS Foundation Trust, Newcastle upon Tyne, UK

**Keywords:** aerosol-generating procedure, dental high-speed equipment, infection control, dental facilities, viral plaque assay, dental disinfectants

## Abstract

Oral microbes are dispersed during dental treatment and reduction methods have been proposed, but dental unit waterline (DUWL) disinfectants have received little attention; specifically, the effect on viruses has not been studied. This study aims to 1) investigate the effect of DUWL disinfectants on viral dispersion in dental bioaerosols and 2) establish a dual-tracer system using live bacteriophage and fluorescein supported by optical particle measurement. Bacteriophage MS2 was used as a viral tracer and fluorescein as a fluorescent tracer. Validation experiments were conducted to exclude interference of one tracer with the other or of DUWL disinfectants on detection methods. Simulated “saliva” containing the tracers was infused into the mouth of a dental mannequin during 10-min dental procedures with an air turbine handpiece (*n =* 3 replicates). Aerosols and droplets were sampled in an enclosed dental operatory using air samplers and settlement onto sterile filter papers. Bacteriophage was quantified using plaque assays and reverse transcription quantitative polymerase chain reaction (RT-qPCR). Fluorescein was quantified fluorometrically. The effect of DUWL disinfectants on total aerosol concentration was assessed in separate experiments using an optical particle counter. DUWL disinfectants reduced bacteriophage viability, and interference between tracers was not observed. In simulated clinical procedures, the disinfectant ICX reduced bacteriophage detection substantially (*P* < 0.001; 2-way analysis of variance). MS2 RNA was detected in all experimental samples but not negative controls. Samples positive on RT-qPCR but not plaque assays may indicate that virions at distant sites are nonviable. Fluorescein tracer showed good agreement with the bacteriophage tracer. DUWL disinfectants designed for continuous presence in irrigants reduce the dispersion of viable virus in dental bioaerosols during simulated procedures. Their use may therefore be important for routine infection control and as a mitigation factor during infectious disease outbreaks. Future studies should explore this using a range of viruses and other microbes.

## Introduction

The oral cavity is one of the body’s most microbially diverse sites, and microorganisms can be dispersed in droplets and aerosols during dental treatment (Innes et al. 2021). Microbes from biofilms in dental unit waterlines (DUWLs) may also be dispersed (Dutil et al. 2007). Disinfection of DUWLs to reduce biofilms is standard practice, but potential dispersion of microbes from the mouth remains. Products are available for periodic DUWL disinfection, which have a residual effect on biofilm accumulation, while other products are intended for continuous use at concentrations safe for patients (Zemouri, de Soet, et al. 2020), and both may influence bacteria and viruses in the mouth.

There has been concern for decades over transmission of pathogens via dental bioaerosols (Micik et al. 1969); perceived importance has fluctuated during several outbreaks, most notably the COVID-19 pandemic (Patton 2021), and much research has focused on bacterial content, with little specific consideration of viruses.

Methods to mitigate dispersion or microbial load, such as dental suction, dental dam, and antimicrobial mouthrinses, have been studied (Samaranayake et al. 2021), but few authors have examined the effect of DUWL disinfectants on microbes contained within dental bioaerosols (Dutil et al. 2007; Sethi et al. 2019). To our knowledge, the effect of these disinfectants on viruses in bioaerosols has not been studied.

The aim of this study is to measure the effect of DUWL disinfectants on the dispersion of viruses in a simulated model of dental bioaerosols. Our secondary aim is to compare measurement of dental bioaerosols using a viral tracer with that of a fluorescent tracer.

## Materials and Methods

### Preparation of Bacteriophage and Fluorescein Tracers

*Escherichia* phage MS2 (*Emesvirus zinderi;* DSM 13767; DSMZ GmbH), a nonenveloped single-stranded RNA virus with ~26-nm diameter virions, was chosen due to its structural similarity to several human viral pathogens (e.g., rhinoviruses, adenoviruses, coronaviruses). MS2 was propagated using *Escherichia coli* (DSM 5695; DSMZ GmbH) cultured on NZCYM agar plates (Table) using the double agar layer method (Ács et al. 2020), whereby 100 µL of bacteriophage and 100 µL of exponential phase *E. coli* were added to 3 mL of soft agar (0.7%) layered over solid agar (1.4%) in a petri dish. Plates were incubated at 37°C until discrete plaques of absent growth were observed in the *E. coli* lawn. Bacteriophage was harvested by adding 3 mL of SM buffer (Table) to plates and rocking for 1 h, before removal of the liquid and top agar. The recovered mixture was centrifuged at 4,000 × *g* for 10 min at 4°C and the supernatant filtered (0.22 μm). Tenfold serial dilutions of bacteriophage suspension in SM buffer were then used in plaque assays as above, and plaques were counted to determine titer (plaque-forming units [PFU]/mL) as follows:

$$\mathrm{PFU}=\frac{N_{\mathrm{plaques}}}{\mathrm{dilution}\;\mathrm{factor}\;\times \;\;\mathrm{volume}\;\left(\mathrm{mL}\right)}.$$

For the fluorescent tracer, fluorescein sodium (Sigma-Aldrich) was added to distilled water (1 g/L).

**Table.** Composition of Media and Buffers Used in This Study.

Medium or Buffer	Reagent	Amount
NZCYM medium	Casein hydrolysate	110 g/L
	Sodium chloride	5 g/L
	Casamino acids	1 g/L
	Yeast extract	5 g/L
	Magnesium sulfate heptahydrate	2 g/L
	Maltose	2 g/L
	Agar	7 – 14 g/L
SM buffer	Sodium chloride	100 mM
	Magnesium sulfate heptahydrate	10 mM
	Tris-HCl	50 mM
	Sodium hydroxide (0.1 M)	To correct to pH 7.5

All reagents were dissolved in deionized water.

### Effects of Disinfectants on Bacteriophage and Fluorescein

Two commonly used DUWL disinfectants with different active ingredients were used: Alpron (Alpro Medical GmbH), containing chloramine-T and polyhexanide biguanide, and ICX (A-dec), containing sodium percarbonate and silver nitrate. These active agents disrupt viral proteins and nucleic acids (McDonnell and Russell 1999), and both disinfectants are intended for continuous use in DUWLs at 1% v/v for Alpron and 106.2 mg/L for ICX.

To determine the effect of DUWL disinfectants on bacteriophage viability, 2-fold serial dilutions of Alpron or ICX (6.25%–100% of manufacturers’ concentrations) and negative controls (no disinfectant) were added in triplicate to MS2 suspension (2 × 10^7^ PFU/mL) and incubated for 10 min at room temperature. The suspension was collected, and viable bacteriophage was quantified by plaque assays.

To determine if ICX interfered with fluorescence readings, it was used at the manufacturer’s concentration to spike 2-fold serial dilutions of fluorescein (0–1 g/L) in triplicate, with incubation for 1 h at room temperature. Fluorescence was then measured.

To determine the effect of fluorescein on bacteriophage viability, 1 g/L fluorescein was added to MS2 suspensions (2 × 10^7^ PFU/mL) and incubated at 4°C for 1 h or 24 h (*n =* 9) before conducting plaque assays. Negative controls were also conducted.

### Simulated Clinical Experiments

Experiments were conducted in a 44.96-m^3^ dental surgery. The room was ventilated via a supply-extract system with ceiling vents, providing 5.0 air changes per hour (ACH). A dental mannequin (P-6/3 TSE; Frasaco GmbH) was attached to a dental chair (A-dec) positioned ~80 cm from the ground. A 2-channel syringe pump (Legato 101; KD Scientific) introduced MS2 (2 × 10^7^ PFU/mL) and fluorescein (1 g/L) suspension into the mannequin’s mouth through two 1-mm internal diameter tubes opening adjacent to the upper incisors at 1.5 mL/min.

Ten-minute crown preparations were performed on the upper right central incisor using an air-turbine dental handpiece (Synea; W&H; irrigant flow rate: 38.3 mL/min). No mitigation technique was used. Two conditions were investigated: 1) water as a handpiece irrigant (control) and 2) ICX at the manufacturer’s recommended concentration (106.2 mg/L) as the irrigant. Three replicates were conducted per experiment.

### Aerosol and Droplet Sampling

**Figure 1.**
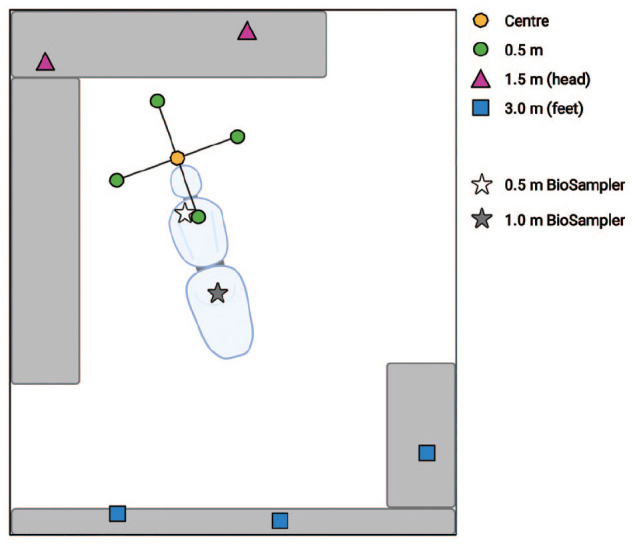
Schematic view of sampling locations in clinical simulation experiments; not to scale. Created using BioRender.com.

To capture settled droplets and aerosols, autoclave-sterilized, 30-mm diameter cotton-cellulose filter papers (Whatman; Cytiva) were positioned at 90° intervals on a rig around the operator (Allison, Dowson, et al. 2021), at a distance of 0.5 m (4 samples per replicate). Filter papers were also placed 1.5 m behind the head of the mannequin (2 samples per replicate), 3.0 m beyond the foot of the dental chair (3 samples per replicate), and 1 filter paper (center) as close as possible to the mouth of the mannequin (Fig. 1).

Suspended aerosols were captured using 2 liquid cyclone air samplers (BioSampler; SKC) located at 0.5 m and 1 m down the length of the dental chair (Fig. 1). BioSamplers were filled with 20 mL of distilled water and operated at 12.5 L/min using a sampling pump (BioLite+; SKC) calibrated using a rotameter (SKC). Sampling began 1 min before the 10-min dental procedure and continued until 30 min after the procedure (41 min total). Negative control samples (1 filter paper and 2 BioSamplers per replicate) were placed in an adjacent, enclosed surgery for the duration of each experiment. Positive control samples of the bacteriophage-fluorescein solution and aspirates from the mannequin’s mouth were collected after each experiment.

To specifically measure the effect of ICX on physical aerosol properties, a separate experiment (*n =* 1) was conducted using an optical particle counter (OPC). Due to room availability, this experiment was conducted in an almost identical, 51.45-m^3^ surgery, in the same clinic as other experiments. To minimize background particle counts and allow measurement, 2 high-efficiency particulate air (HEPA) filter devices (DA-UVC1001; VODEX), together contributing 11.66 equivalent ACH, supplemented the 3.30 ACH provided by the central ventilation. This additional measure was not used in bacteriophage experiments to replicate more realistic clinical conditions. An air-turbine dental handpiece (Synea; W&H; 22 mL/min) was positioned with the bur tip 1 cm below the upper right central incisor tooth of a mannequin and operated in a steady state without tooth contact for 30 min per experiment. This allowed controlled measurement of aerosol without added variability of a dental procedure; this was repeated under 3 conditions: 1) water irrigant, 2) ICX at the manufacturer’s concentration (106.2 mg/L; 1× ICX), and 3) ICX at 10 times the manufacturer’s concentration (1.062 g/L; 10× ICX). A single 30-min replicate of each condition was performed, with no other mitigation, which is equivalent to three 10-min replicates in other experiments.

A laser-diode OPC (3016-IAQ; Lighthouse) with 6 particle-size channels (0.3–10.0 µm) and a flow rate of 2.83 L/min was positioned 30 cm inferior to and 30 cm above the plane of the mannequin’s mouth. Sampling began 10 min before operating the handpiece (40 min total). Particle number concentration was calculated by summing particle counts per cm^3^ across all particle size bins (further details in the Appendix).

### Detection of Bacteriophage and Fluorescein Tracers

Filter papers were washed using 350 μL of deionized water, vortexed, and centrifuged (Allison, Currie, et al. 2021) to elute fluorescein and bacteriophage. BioSampler media were analyzed directly. To quantify fluorescein, 100 µL of eluate was transferred to a 96-well microtiter plate in duplicate and fluorescence measured in relative fluorescence units (RFU) using a Synergy HT microplate reader (BioTek) (Allison, Currie, et al. 2021).

Plaque assays were performed (Ács et al. 2020) to quantify viable bacteriophage. Plates were incubated at 37°C for 7 h and plaques counted. Filter paper surface area (7.07 cm^2^) was used to calculate RFU/cm^2^ and PFU/cm^2^.

### Reverse Transcription Quantitative Polymerase Chain Reaction

Bacteriophage RNA was extracted from 50 µL of eluate using the GeneJET Viral DNA/RNA Purification Kit (Thermo Fisher Scientific). Supplied wash buffers were supplemented with >99% ethanol (Thermo Fisher Scientific). The supplied carrier RNA and proteinase K were used to supplement lysis solution according to the manufacturer’s instructions. RNA was eluted in 50 µL of the elution buffer and stored at −80°C or immediately reverse transcribed using a high-capacity complementary DNA (cDNA) reverse transcription kit (Applied Biosystems) according to the manufacturer’s instructions before immediate reverse transcription quantitative polymerase chain reaction (RT-qPCR) or storage for up to 10 d at −80°C.

MS2 primers and hydrolysis probe (Gendron et al. 2010) were supplied by Thermo Fisher Scientific. Premix ExTaq for probe-based qPCR (Takara) was used in all reactions. Primers and probe were used at 1-μM and 2-μM concentrations, respectively, with 2 μL of cDNA template. No-template and no-reverse-transcriptase controls were included in each reaction. A QuantStudio 3 thermal cycler (Thermo Fisher Scientific) was used as follows: 30-s initial denaturation at 95°C, then 95°C for 5 s and 57°C for 30 s, for 40 cycles. PCR products were evaluated using agarose gel electrophoresis to confirm PCR product size. Samples that amplified within 40 cycles were classified as positive for MS2 RNA.

### Statistical Methods

SPSS Statistics 27 (SPSS, Inc.) and GraphPad Prism 9 (GraphPad Software) were used for analysis. Normality was assessed using quantile–quantile plots and Shapiro–Wilk tests. One-way analysis of variance (ANOVA) with post hoc Dunnett T3 was used to assess the effect of Alpron and ICX on MS2 viability, and Spearman’s ρ and associated *P* were calculated. Two-way ANOVA was used to examine the effect of distance from the procedure and the presence of ICX on the detection of bacteriophage and fluorescein. Mean particle number concentration was compared across OPC experiments using 1-way ANOVA with post hoc Dunn’s test, and α = .05 for all tests.

## Results

### Optimization of Bacteriophage-Fluorescein Tracer

**Figure 2.**
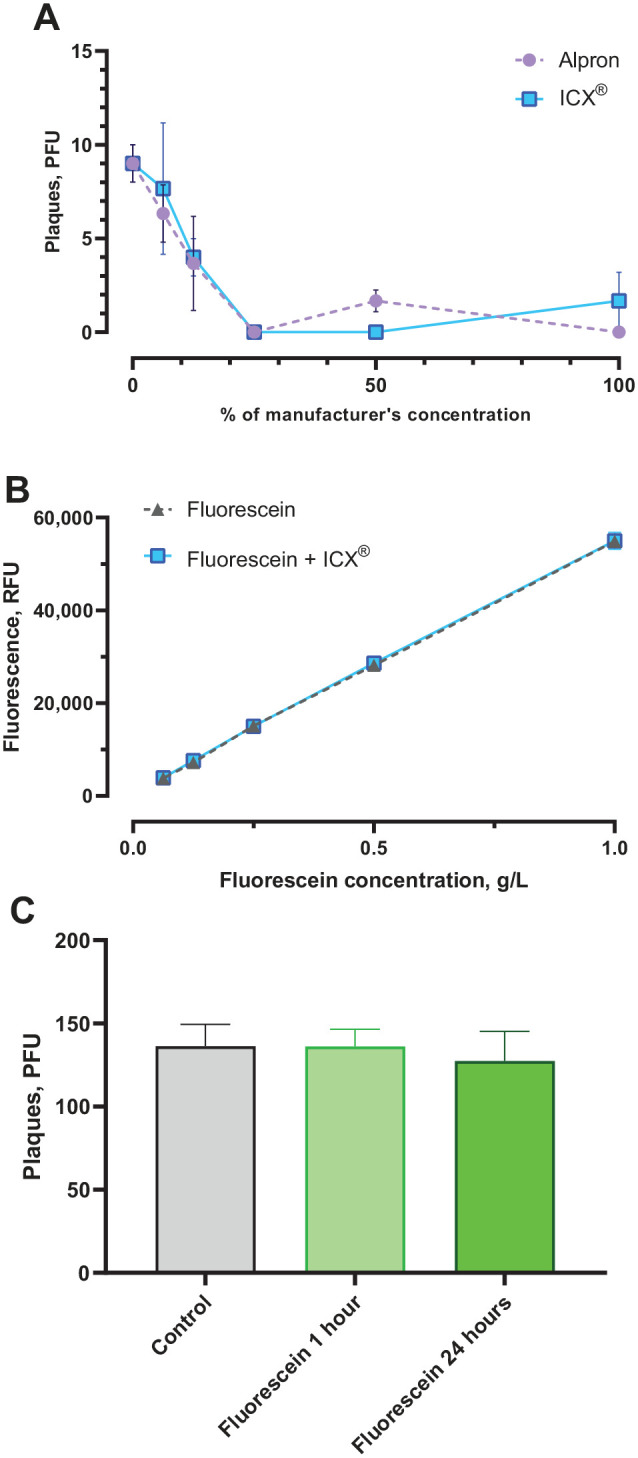
Results of laboratory validation experiments. (**A**) Effect of Alpron and ICX on viable MS2 bacteriophage detected by plaque assay (*n =* 3 per data point). (**B**) Effect of ICX at the manufacturer’s concentration on fluorescence from fluorescein (*n =* 3 per data point). *R*^2^ = .999 for both fluorescein and fluorescein + ICX. (**C**) Effect of fluorescein on viable MS2 bacteriophage detected by plaque assays after incubation with 1 g/L fluorescein for 1 h or 24 h (*n =* 9 per condition). Error bars show standard deviation. PFU, plaque-forming units; RFU, relative fluorescence units.

Alpron and ICX reduced MS2 plaque counts following 10-min incubation compared to control (Fig. 2A); this was statistically significant for concentrations above 6.25% of the manufacturer’s concentration for Alpron and above 12.5% for ICX (all *P <* .05). Plaque counts were highly negatively correlated with disinfectant concentration (Alpron ρ = −.802, *P <* .001; ICX ρ = −.848, *P <* .001).

Fluorescence from ICX-spiked fluorescein was not statistically significantly different from nonspiked fluorescein, accounting for dilution (2-way ANOVA, main effects for ICX: *F*(4, 30) = .452, *P* = .509; Fig. 2B). Fluorescein concentration showed a linear relationship with fluorescence readings for both ICX-spiked (*R*^2^ = .999) and nonspiked fluorescein (*R*^2^ = .999).

Plaque counts did not differ between cultures incubated with fluorescein for 1 h, 24 h, and controls (1-way ANOVA, *F*(2, 24) = 1.143, *P =* .336; Fig. 2C).

### Effect of ICX on Bacteriophage in Simulated Clinical Procedures

**Figure 3.**
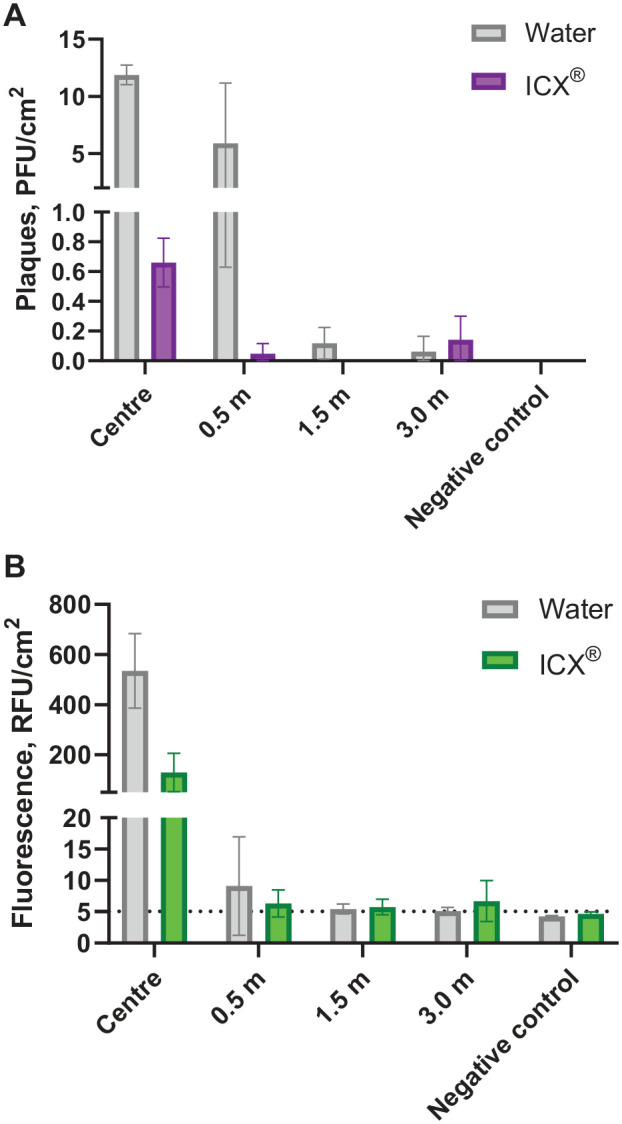
Results of clinical simulation experiments. Effect of ICX in dental handpiece irrigant on (**A**) viable MS2 bacteriophage tracer recovered from filter paper samples and detected by plaque assay and (**B**) fluorescein tracer recovered from filter papers and detected fluorometrically. *n* = 3 replicates were conducted for each experimental condition, and multiple samples were present at each distance from the procedure, thereby giving the following number of samples per condition at each distance: 0.5 m, *n =* 12; 1.5 m, *n =* 6; 3.0 m, *n =* 9; center and negative control, *n =* 3. Error bars show standard deviation. Dotted line shows lower limit of detection for fluorescein (negative control mean + 2 SD). Normalized for surface area of filter papers used for collection (7.07 cm^2^). PFU, plaque forming units; RFU, relative fluorescence units.

ICX was chosen for clinical experiments due to its routine use in the study setting. Experiments examining bacteriophage detection in aerosols and droplets showed substantially reduced dispersion with ICX (2-way ANOVA, *F*(1, 60) = 35.7, *P <* .001, η2p = .407). Detection reduced with increasing distance from the procedure (*F*(3, 60) = 13.6, *P <* .001, η2p = .440), with a significant interaction of the 2 factors (*F*(3, 60) = 12.1, *P <* .001, η2p = .411). A small amount of viable bacteriophage was detected in the 0.5- and 1.0-m BioSamplers during ICX experiments (1.17 and 0.26 PFU/L_air_, respectively) but not during experiments with water. The reason for this is unclear but is likely due to the unavoidably variable nature of the simulated clinical procedure. Viable bacteriophage was not detected in negative control samples. Highest plaque counts were obtained from central filter papers (Water mean [SD] = 11.88 PFU/cm^2^ [0.85]; ICX = 0.66 PFU/cm2 [0.16]) with little detected at 1.5 m or 3.0 m (Fig. 3A).

MS2 RNA was detected in by RT-qPCR all filter paper samples and BioSamplers in experiments with both water and ICX. Positive control samples (bacteriophage-fluorescein tracer and mannequin mouth aspirate) were positive for MS2 RNA, and all negative control samples were negative.

### Effect of ICX on Fluorescein in Simulated Clinical Procedures

ICX had a statistically significant effect on fluorescein detection in aerosols and droplets, with lower detection when ICX was used (2-way ANOVA, *F*(1, 60) = 108.7, *P <* .001, η2p = .676). Detection reduced with increasing distance from the procedure (*F*(3, 60) = 173.9, *P <* .001, η2p = .909), with a significant interaction of the 2 factors (*F*(3, 60) = 67.6, *P <* .001, η2p = .796). Fluorescein measurements from BioSamplers were not significantly different from negative controls during water and ICX experiments. Highest fluorescence readings were obtained from central filter papers (Water mean [SD] = 534.38 RFU/cm^2^ [148.79]; ICX = 128.93 RFU/cm2 [76.64]) with low readings at 0.5 m onward (Fig. 3B).

### Effect of ICX on Physical Aerosol Properties

**Figure 4.**
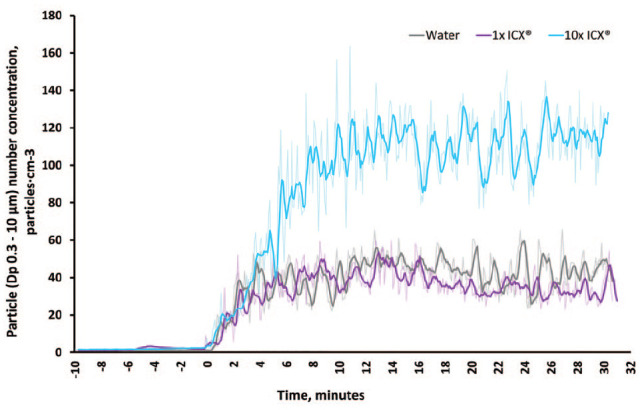
Time-series data of particle number concentration (D_p_ 0.3–10.0 µm) for each experimental condition in optical particle counter experiments. 1× ICX = ICX at manufacturer’s concentration; 10× ICX = ICX at 10 times manufacturer’s concentration; D_p_, particle diameter. The air-turbine handpiece was operated continuously beginning at 0 min. Sampling interval = 5 s shown by feint line; bold line shows 5-point moving average.

In OPC experiments, mean particle number concentration was lower when ICX was used at the manufacturer’s concentration (mean difference = 5.84 particles/cm^3^; 95% confidence interval [CI], 3.96–7.72; *P <* 0.0001), but time-series data overlap substantially with that of water (Fig. 4), suggesting there is no clinically meaningful difference. Mean particle number concentration was substantially higher for ICX at 10 times the manufacturer’s concentration (mean difference = 69.17 particles/cm3; 95% CI, 67.10–71.24; *P <* 0.0001). Further details are given in the Appendix.

## Discussion

In this study, ICX and Alpron reduced MS2 viability in solution in laboratory experiments, consistent with their effect on bacteria in DUWL biofilms (Zemouri, de Soet, et al. 2020). In clinical experiments, ICX in instrument irrigation solutions significantly reduced dispersion of virus in aerosols and droplets, and as confirmed by OPC, this effect does not appear to be because of a reduction in the total amount of aerosol at normal concentrations. This is of clinical importance and means that disinfectants commonly used to control DUWL biofilms, which are designed to be used continuously in irrigant solutions, have the potential to reduce dispersion of pathogens from the mouth within aerosols and droplets. This measure may therefore serve as an important mitigation factor during infectious disease outbreaks (such as the COVID-19 pandemic) and is likely already in place in many dental settings. This may also be an important routine infection prevention and control measure to reduce aerosolization of oral commensal organisms.

MS2 and fluorescein can be successfully used together as tracers to measure bioaerosols, which may be useful in applications elsewhere in and outside of health care, where it is desirable to measure biological and nonbiological properties of bioaerosols. The tracers show good agreement, and fluorescein is therefore a pragmatic tracer where the added complexity of a viral tracer is undesirable; this supports the findings of previous studies using fluorescent tracers (Harrel et al. 1998; Kaufmann et al. 2020; Llandro et al. 2021).

Reduced detection of fluorescein at the central location with ICX was surprising, as laboratory experiments showed fluorescence is not affected, and OPC experiments show that ICX does not affect the amount of aerosol produced at normal concentrations. Relative reduction by ICX was much smaller for fluorescein compared to bacteriophage, and this finding was only present at this location for fluorescein but also occurred at 0.5 m for the bacteriophage tracer. It is possible that the difference in fluorescein detection was due to the inherent variability of the dental procedure. An interesting finding was that at 10 times normal concentration, ICX increased aerosol number concentration; the mechanism behind this is unclear, but it may that the presence of concentrated surfactants affects particle size and therefore increases particle number concentration.

“Saliva” was dispersed from the mouth in our model of dental bioaerosols, with most contamination falling as droplets and settled aerosols within 0.5 m; lower contamination occurred at greater distances (at least 3 m). Low levels of dispersed “saliva” were seen in suspended aerosols, and this decreased with distance from the source. These findings agree with studies using similar and complementary methodologies (Zemouri, Volgenant, et al. 2020; Vernon et al. 2021). Bacteriophage was detected in suspended aerosols in a single experimental condition, which is likely due to the inherent variability of the clinical procedure and the stochastic interaction of “saliva” and the aerosols produced by dental instruments.

All experimental samples were positive for bacteriophage RNA using RT-qPCR, indicating that this method may be more sensitive than plaque assays, consistent with others’ work (Ács et al. 2020), or that at distant sites viral particles are not viable and are therefore not detected in plaque assays. MS2 RNA was not detected in any negative control samples, providing confidence in these findings.

This study has several important limitations. First, this is an in vitro model, and the inherent variability of patient anatomy, salivary flow rates, viral load, instrument irrigation flow rates, and other mitigation measures are not accounted for. However, we chose to use realistic parameters (e.g., salivary viral load [Yang et al. 2021] and flow rate [Ship et al. 1991]), and standardization of the model across replicates and experimental conditions allows us to address the research question in a controlled manner. Similarly, the inherent antimicrobial properties of saliva (Vila et al. 2019) were not accounted for, and saliva may reduce dispersion of pathogens in bioaerosols in vivo, reducing the true effect of the intervention in this study. Second, it is possible that losses in recovery efficiency are introduced during elution of bacteriophage from filter papers; we elected to use this method as opposed to placing agar plates in the clinical environment (Vernon et al. 2021), so that eluted bacteriophage may be diluted to allow quantification of higher plaque counts than is possible with agar plates. In addition, elution from filter papers allows downstream RT-qPCR. Finally, this study shows that ICX reduces MS2 dispersion in aerosols, but other viruses may not necessarily behave in the same way. Only 1 other bacteriophage, *Pseudomonas virus phi6* (Vernon et al. 2021), has been investigated in simulated dental bioaerosols in clinical settings, and in nonclinical dental simulations, 2 studies have used other human (Ionescu et al. 2021) and equine viruses (Fidler et al. 2021). There therefore remains a need to examine the behavior of different viruses in bioaerosols and those that are clinically relevant.

## Conclusion

DUWL disinfectants reduce dispersion of viable viruses in dental bioaerosols; their use is therefore an important mitigation factor for routine infection prevention and control, as well as for dental care provision during infectious disease outbreaks. It is possible to combine a viral tracer with a fluorescent tracer to study bioaerosols, which may be of use in this and other fields of bioaerosol research. Future studies should look at a range of viruses and other microbes in dental bioaerosols to understand variation across different organisms.

## Supplemental Material

sj-docx-1-jdr-10.1177_00220345221093522 – Supplemental material for Waterline Disinfectants Reduce Dental Bioaerosols: A Multitracer Validation Click here for additional data file. Supplemental material, sj-docx-1-jdr-10.1177_00220345221093522 for Waterline Disinfectants Reduce Dental Bioaerosols: A Multitracer Validation by J.R. Allison, C. Dowson, N.S. Jakubovics, C. Nile, J. Durham and R. Holliday in Journal of Dental Research

## Author Contributions

J.R. Allison, R. Holliday, contributed to conception, design, data acquisition, analysis, and interpretation, drafted and critically revised the manuscript; C. Dowson, contributed to design, data acquisition, analysis, and interpretation, drafted and critically revised the manuscript; N.S. Jakubovics, contributed to conception, design, data analysis, and interpretation, drafted and critically revised the manuscript; C. Nile, contributed to design, data interpretation, critically revised the manuscript; J. Durham, contributed to data interpretation, critically revised the manuscript. All authors gave final approval and agree to be accountable for all aspects of the work.
