# Supplementary material for: Waterline Disinfectants Reduce Dental Bioaerosols: A Multitracer Validation
Source: J Dent Res. 2022 May 1;101(10):1198–204. doi: 10.1177/00220345221093522 (PMC9397394; doi:10.1177/00220345221093522)
Supplement: sj-docx-1-jdr-10.1177_00220345221093522 – Supplemental material for Waterline Disinfectants Reduce Dental Bioaerosols: A Multitracer Validation [file sj-docx-1-jdr-10.1177_00220345221093522.docx]

**Waterline disinfectants reduce dental bioaerosols—a multi-tracer validation**

JR Allison, C Dowson, NS Jakubovics, C Nile, J Durham, R Holliday

**Supplementary Appendix**

**Methods**

*Measurement of physical aerosol properties using an optical particle counter*

To measure the effect of ICX^®^ on the physical properties of aerosols produced by the air-turbine handpiece, separate experiments were conducted using an optical particle counter to measure aerosol particles between 0.3 – 10 µm.

Experiments were conducted in a 51.45 m^3^ (5.27 [l] x 3.67 [w] x 2.66 [h] m) enclosed single dental surgery at Newcastle Dental Hospital, Newcastle upon Tyne Hospitals NHS Foundation Trust. The room was ventilated via a supply-extract, centralised Heating, Ventilation, and Air Conditioning (HVAC) system, providing 3.30 air changes an hour. HVAC ventilation was supplemented with two High-Efficiency Particulate Air (HEPA) filtration units (DA-UVC1001; VODEX Ltd., UK), each providing a nominal clean-air delivery rate of 300 L/min, together contributing 11.66 equivalent ACH. This equates to a total equivalent rate of 14.96 ACH assuming homogenous airflow.

A dental mannequin (P-6/3 TSE, Frasaco GmbH; Germany) with model teeth (Frasaco GmbH; Germany) was attached to a dental chair (A-dec Inc.; USA) positioned approximately 80 cm from the ground. An air-turbine dental handpiece (Synea, W&H; Austria; irrigant flow rate: 22 mL/min) with a tapered diamond bur was positioned with the tip of the bur 1 cm below the incisal edge of the upper right central incisor tooth. No dental suction or other mitigation technique was used during experiments. The handpiece was run continuously for 30 min per experiment, under three experimental conditions: 1) experiments with plain water as the irrigant (control); 2) ICX^®^ at the manufacturer’s recommended concentration of 106.2 mg/L (1x ICX^®^) as the irrigant; 3) ICX^®^ at ten times the manufacturer’s recommended concentration (1.062 g/L; 10x ICX^®^) as the irrigant. A single replicate of each condition was performed.

A laser-diode optical particle counter (OPC; 3016-IAQ, Lighthouse; USA) was used to measure aerosols from the handpiece irrigant. The OPC has six particle-size channels (0.3, 0.5, 1.0, 2.5, 5.0, and 10.0 µm) with a sampling flow rate of 2.83 L/min, calibrated to ISO 21501-4 by the manufacturer. The OPC was positioned 30 cm above the plane of the handpiece, and 30 cm inferior to the mouth of the mannequin. Sampling began at 10 min before the handpiece was operated and throughout the 30-min experiment (40 min sampling total). HEPA filtration units were run for at least 20 min before the first experiment, and for at least 1 hour between experiments to ensure particle counts returned to baseline.

Particle number concentration was calculated by summing particle counts per cm^3^ across all particle size bins. This was plotted as a time series for each experiment and with a 5-period moving average. To calculate mean particle number concentration, only data after particle counts had reached a steady state were included based on the time series data. To compare mean particle number concentration across experimental conditions ANOVA was used with *post hoc* Dunn’s test.

**Results**

*Effect of ICX^®^ on physical aerosol properties*

Background particle readings on the day of OPC experiments were 1.55 particles(D_p_ 0.3 – 10.0 µm)/cm^3^ (SD: 1.09) whilst HEPA devices were in use.

Mean particle number concentration was lower for 1x ICX^®^ than for water with a mean difference of 5.84 particles/cm^3^ (95%CI: 3.96 – 7.72; *p* < 0.0001; Appendix Figure 1). The time series data for these groups overlap substantially, suggesting that there is no clinically meaningful difference in the amount of aerosol produced by the air-turbine with these two irrigants (Figure 4, main paper). Mean particle number concentration was substantially higher for 10x ICX^®^ than for water with a mean difference of 69.17 particles/cm^3^ (95%CI: 67.10 – 71.24; *p* < 0.0001; Appendix Figure 1). The time series data for the 10x ICX^®^ was markedly separated from the other two groups (Figure 4, main paper), suggesting a clinically meaningful difference in aerosol production for the 10x ICX^®^ group.

Visual assessment of particle size distribution across the three experimental groups does not indicate a substantial difference (Appendix Figure 2).

**Appendix Figure 1.** Particle number concentration (D_p_ 0.3 – 10.0 µm) for each experimental condition. Data taken from when particle concentration reached a discernible plateau (Figure 4, main paper): 6 min for Water and 1x ICX^®^; 12 min for 10x ICX^®^. 1x ICX^®^ = ICX^®^ at manufacturer’s concentration; 10x ICX^®^ = ICX^®^ at ten times manufacturer’s concentration; D_p_ = particle diameter. **** = *p* < 0.0001 (One-way ANOVA with *post hoc* Dunn’s test).


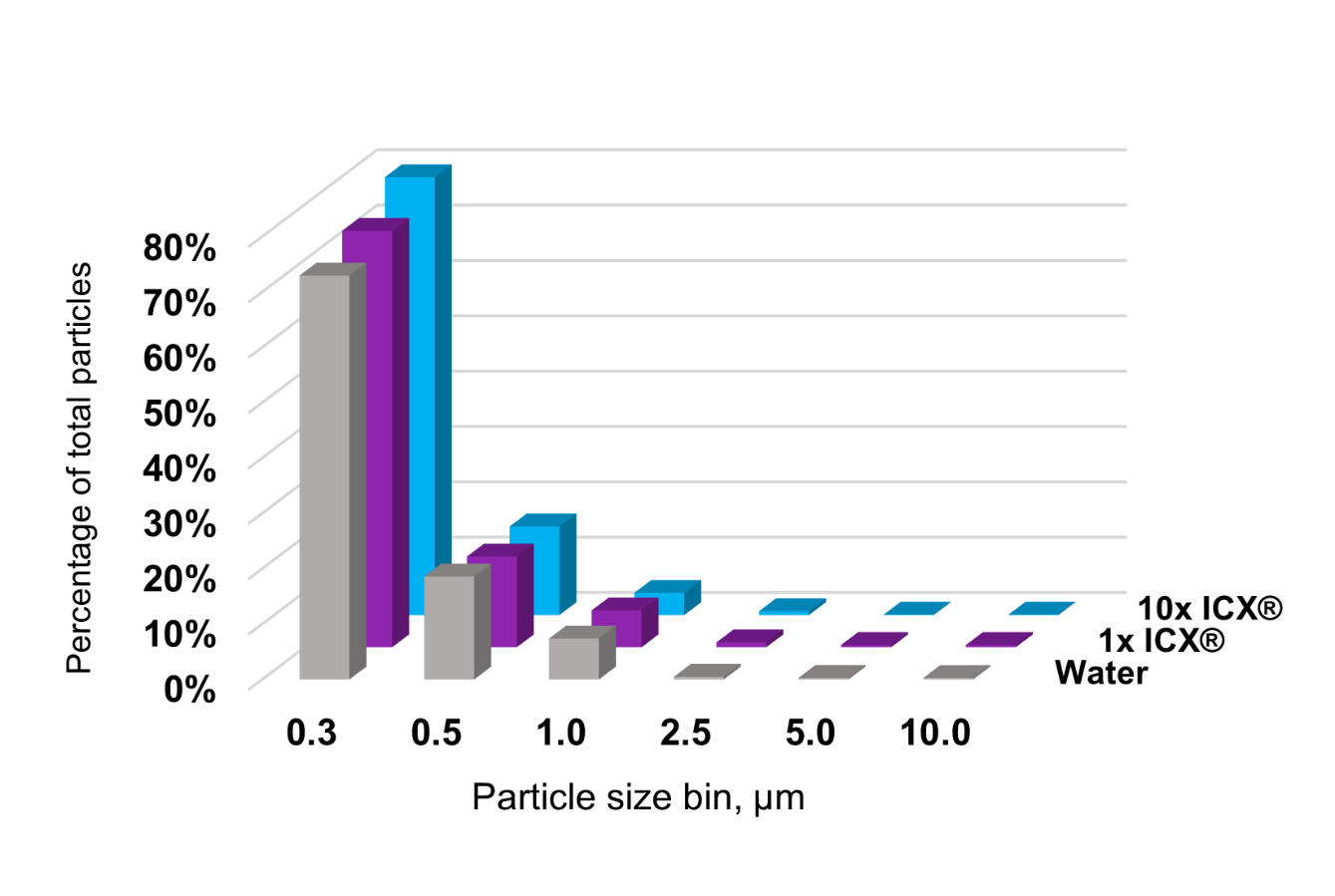


**Appendix Figure 2.** Particle size distribution as percentage of total particles detected for each particle size bin across each experimental condition. Data taken from min 12 – 30 as particle concentration was stable at this time for all experimental conditions. 1x ICX^®^ = ICX^®^ at manufacturer’s concentration; 10x ICX^®^ = ICX^®^ at ten times manufacturer’s concentration.
